# Functional Maps of Human Auditory Cortex: Effects of Acoustic Features and Attention

**DOI:** 10.1371/journal.pone.0005183

**Published:** 2009-04-13

**Authors:** David L. Woods, G. Christopher Stecker, Teemu Rinne, Timothy J. Herron, Anthony D. Cate, E. William Yund, Isaac Liao, Xiaojian Kang

**Affiliations:** 1 Human Cognitive Neurophysiology Laboratory, VANCHCS, Martinez, California, United States of America; 2 Department of Neurology, University of California Davis, Sacramento, California, United States of America; 3 Center for Neurosciences, University of California Davis, Davis, California, United States of America; 4 Center for Mind and Brain, University of California Davis, Davis, California, United States of America; 5 Department of Speech and Hearing Sciences, University of Washington, Seattle, Washington, United States of America; 6 Department of Psychology, University of Helsinki, Helsinki, Finland; University of Granada, Spain

## Abstract

**Background:**

While human auditory cortex is known to contain tonotopically organized auditory cortical fields (ACFs), little is known about how processing in these fields is modulated by other acoustic features or by attention.

**Methodology/Principal Findings:**

We used functional magnetic resonance imaging (fMRI) and population-based cortical surface analysis to characterize the tonotopic organization of human auditory cortex and analyze the influence of tone intensity, ear of delivery, scanner background noise, and intermodal selective attention on auditory cortex activations. Medial auditory cortex surrounding Heschl's gyrus showed large sensory (unattended) activations with two mirror-symmetric tonotopic fields similar to those observed in non-human primates. Sensory responses in medial regions had symmetrical distributions with respect to the left and right hemispheres, were enlarged for tones of increased intensity, and were enhanced when sparse image acquisition reduced scanner acoustic noise. Spatial distribution analysis suggested that changes in tone intensity shifted activation within isofrequency bands. Activations to monaural tones were enhanced over the hemisphere contralateral to stimulation, where they produced activations similar to those produced by binaural sounds. Lateral regions of auditory cortex showed small sensory responses that were larger in the right than left hemisphere, lacked tonotopic organization, and were uninfluenced by acoustic parameters. Sensory responses in both medial and lateral auditory cortex decreased in magnitude throughout stimulus blocks. Attention-related modulations (ARMs) were larger in lateral than medial regions of auditory cortex and appeared to arise primarily in belt and parabelt auditory fields. ARMs lacked tonotopic organization, were unaffected by acoustic parameters, and had distributions that were distinct from those of sensory responses. Unlike the gradual adaptation seen for sensory responses, ARMs increased in amplitude throughout stimulus blocks.

**Conclusions/Significance:**

The results are consistent with the view that medial regions of human auditory cortex contain tonotopically organized core and belt fields that map the basic acoustic features of sounds while surrounding higher-order parabelt regions are tuned to more abstract stimulus attributes. Intermodal selective attention enhances processing in neuronal populations that are partially distinct from those activated by unattended stimuli.

## Introduction

Neurophysiological studies have elucidated the functional organization of auditory cortex in non-human primates by mapping tonotopically organized auditory cortical fields (ACFs) and then evaluating the functional specialization of neurons in selected tonotopic and non-tonotopic regions to different stimulus features and task parameters [1]. Such studies are possible because neurophysiological recordings can be obtained in multiple experimental sessions in the same monkey. In contrast, fMRI studies of the tonotopic organization of human auditory cortex have typically imaged subjects in a single experimental session [2]. Although other studies have characterized the effects of sound intensity [3], ear of delivery [4], scanner masking noise [5], and selective attention [6] on activations in auditory cortex, the influence of these variables on tonotopic and non-tonotopic ACFs is not yet fully understood. The goal of the current experiment was to clarify the functional organization of human auditory cortex by mapping its tonotopic organization while simultaneously manipulating the intensity, spatial location and attentional relevance of auditory signals.

The first objective of the current study was to visualize the basic tonotopic organization of human auditory cortex. Previous fMRI studies using tones or narrow-band noise bursts have consistently identified two frequency-specific regions: a posterior-medial high-frequency region and a more anterior-lateral low-frequency region [7]–[10]. In addition, studies using cortical surface mapping techniques have identified additional frequency-specific fields in individual subjects. For example, Talavage and colleagues mapped auditory cortex in the left hemisphere with surface coils and narrow-band noise bursts [11] and continuously changing frequency sweeps [12]. They found eight frequency-specific regions, four tuned to high frequencies and four tuned to low frequencies. Among the eight fields were two regions with properties similar to those reported in previous studies – a medial high-frequency region that was located in Heschl's sulcus (HS) and a low-frequency region located on mid-Heschl's gyrus (HG). They also reported a third high-frequency region anterior to HG.

Formisano and colleagues [2] mapped activations on the surface of the left hemisphere in six subjects using surface coils and sparse image acquisition in a 7 T scanner. They found evidence for two mirror-symmetric tonotopic maps encompassing the aforementioned three frequency-specific regions – the first connected a high-frequency region in HS with a low-frequency region in mid-HG and the second connected the same low-frequency region with a high-frequency region near the junction of HG and the superior temporal gyrus (STG). These results suggested that human auditory cortex conforms to the general primate model with two mirror-symmetric tonotopic maps in primary auditory core and belt areas that are joined at a common low-frequency boundary [13], [14].

One issue left unresolved by previous studies of the tonotopic organization of human auditory cortex is the influence of hemispheric specialization. Although most whole-brain studies have suggested that the two hemispheres differ in tonotopic organization, the results are inconsistent. Some studies report tonotopic organization primarily in the left hemisphere [7], [10], [15] while others report it primarily in the right hemisphere [16], [17]. Although the cause of these inconsistencies remains unknown, inter-hemispheric differences in auditory cortex organization might be expected because of hemispheric asymmetries in auditory cortex anatomy [18], [19], hemispheric asymmetries in the magnitude of sensory responses elicited by sounds [20], and the well-established functional specialization of the left hemisphere for language processing [21].

Most previous fMRI studies of tonotopic organization have not examined the effect of sound intensity on frequency maps. The spatial extent of activations in auditory cortex increase when sounds become more intense [16], [22]–[24]. These increases in activation extend are thought to reflect the in width of neuronal frequency tuning curves at increased sound intensities [25] so that louder sounds activate neurons tuned to a larger range of sound frequencies. This suggests that lower intensity sounds should excite more frequency-specific regions of auditory cortex [8]. The distribution of activations might also be expected to show subtle shifts with intensity because the bandwidth of neuronal intensity coding properties is non-randomly distributed in isofrequency bands within A1 [26]. Using fMRI, Bilecen and colleagues found evidence of such an effect [3] – a lateral to medial shift in activation foci as sound intensities increased.

Sound location also has a major influence on the magnitude and extent of activations in auditory cortex. For example, monaural sounds produce activations that are significantly larger in the hemisphere contralateral to stimulation [4], [27]–[31]. However the effects of binaural stimulation are less consistent. Some studies find that activations produced by binaural sounds are similar to those produced by contralateral monaural sounds [4] whereas others find that binaural responses are intermediated in amplitude between those produced by contralateral and ipsilateral sounds [32]. Moreover, binaural cues that are used to analyze sound motion may be preferentially processed in the right hemisphere [33].

Another important variable in fMRI studies of auditory cortex is the acoustic noise that accompanies image acquisition. This problem has led to the development of “sparse” image acquisition protocols in which images are acquired infrequently while sounds are delivered during the relatively silent intervals between image acquisitions [34], [35]. Sparse imaging has been used in many recent fMRI investigations of tonotopic organization [2], [8], [13], [36], [37]. However, the extent to which image acquisition parameters modify functional maps in auditory cortex remains to be determined. Although continuous imaging may alter the distribution of activations by differentially masking certain sound frequencies [38], [39], continuous imaging has been used successfully in many studies of tonotopic organization [7], [11], [12] and it is critical for analyzing the temporal properties that may distinguish different auditory processing operations [28], [31], [40], [41].

While many studies have demonstrated enhanced activations in auditory cortex when subjects actively attend to auditory signals [6], [28], [42]–[47] the nature of these attention effects remains incompletely understood. One possibility is that attention simply enhances sensory responses in a manner analogous to increasing their signal-to-noise ratio or intensity [48], [49]. This hypothesis suggests that attention-related modulations (ARMs) should have distributions and functional properties similar to those of sensory responses themselves. For example, attending to tones of a particular frequency should enhance activations in corresponding frequency-selective regions of auditory cortex [50]. Alternatively, attention may preferentially engage non-tonotopic fields that process more abstract auditory stimulus properties and have distinct functional properties [1], [28].

A central problem in visualizing the organization of human auditory cortex has been the technical challenge of creating average cortical surface maps for a subject population. Studies of tonotopic organization have revealed that the anatomical locations of frequency-specific regions have a coarse but consistent relationship to local anatomical landmarks in individual subjects [2]. This consistency implies that it should be possible to image the average tonotopic organization in a subject population provided that the anatomical features of auditory cortex can be accurately aligned across the subject population. In a previous study we used local-landmark mapping [51] to align the auditory cortical surface across subjects and found reliable tonotopic organization in population averages [28]. However, local-landmark methods require anatomical fiducial points to be manually identified in each subject. As a result, average activation maps are not uniquely and objectively determined for a subject population. Recent reports have shown that cortical sensory areas, including auditory cortex [43], [52], can be accurately aligned across subject populations using objective, whole-brain cortical-surface alignment [53]. In the current experiment we used whole-brain cortical-surface alignment to characterize the average functional organization of auditory cortex in a group of normal young subjects.

## Results

In the current study each subject participated in six functional brain imaging sessions of an intermodal selective attention task that independently manipulated attention as well as tone frequency, intensity, ear of delivery, and image acquisition parameters (sparse vs. continuous imaging). The randomized factorial design permitted the isolation of consistent activation patterns associated with particular acoustic features across a wide range of variation of other stimulus and task parameters. For example, tonotopic organization was analyzed to tone patterns of different frequency across 36 different task conditions (two tone intensities, three tone locations, continuous vs. sparse image acquisition, and three attention conditions).

**Figure 1:**
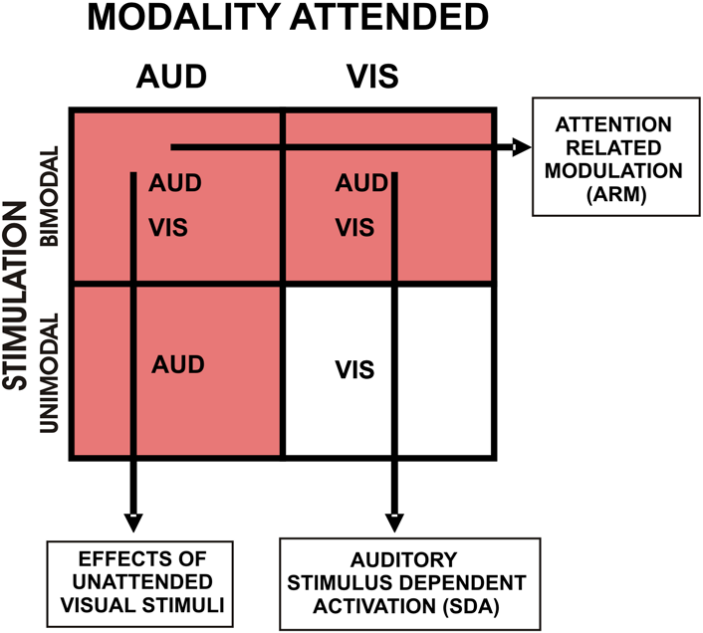
Conditions in the intermodal selective attention experiment. Red blocks show conditions with auditory stimuli. Arrows show the subtractions used for the analysis of sensory responses and attention-related modulations in auditory cortex. SDA = stimulus dependent activation occurring in the absence of auditory attention. ARM = attention-related modulations.

**Figure 2:**
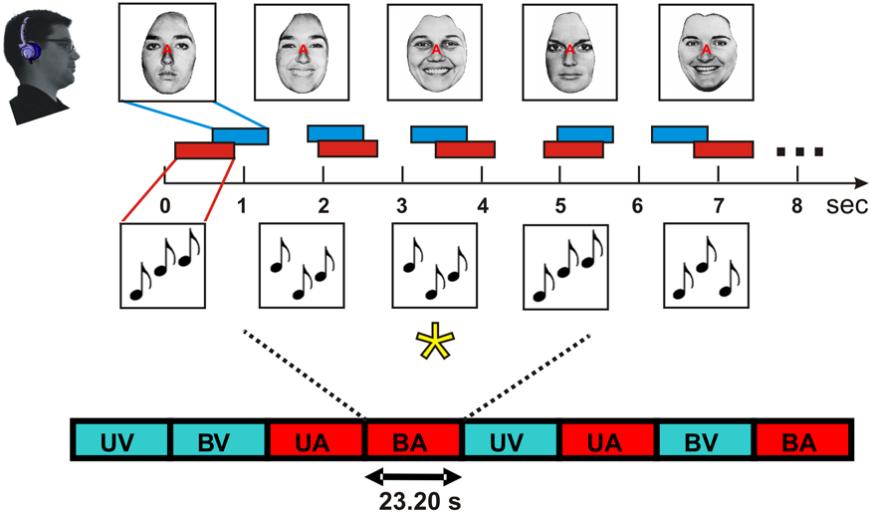
Intermodal selective attention paradigm. Stimuli were presented in blocks lasting 23.2 s. Auditory stimuli were three-tone patterns varying in intensity (90 or 70 dB SPL), location (left ear, right ear or binaural) and frequency (center frequency = 225 Hz, 900 Hz, or 3600 Hz) in different blocks. Visual stimuli were pictures of faces or words. Subjects focused attention on the modality cued by the letter at fixation (e.g., “A” top) and performed difficult one-back matching task matching repeated tone patterns during auditory attention ( target = asterisk). Auditory and visual stimuli were presented with randomized asynchronous onsets to minimize multimodal integration. Attend-auditory (red) and attend-visual (blue) blocks occurred in constrained random order. UV = unimodal visual. UA = unimodal auditory. BV = bimodal, visual attention. BA = bimodal, auditory attention.

Stimuli were presented in blocks that contained unimodal visual (UV), unimodal auditory (UA), or bimodal stimuli. In bimodal blocks, either auditory (BA) or visual (BV) stimuli could be attended. Subjects always attended to a single modality that was randomly assigned on each block (Figure 1). A comparison of activations in unimodal and bimodal blocks permitted the isolation of stimulus-dependent activations (SDAs), defined as the difference between activations in unimodal and bimodal blocks caused by the addition of unattended stimuli: i.e., auditory SDAs were obtained from BV-UV subtractions. Attention-related modulations (ARMs) were isolated from BA-BV subtractions: i.e., from blocks containing the same stimuli during auditory and visual attention conditions. Additionally, All-Auditory Stimulation Activations (All-ASAs) were computed by subtracting unimodal visual blocks (UV) from the three blocks containing auditory stimulation (UA, BA and BV). The attended modality was cued by a partially transparent letter (“A” or “V”) at fixation, and subjects performed a difficult one-back matching task in the attended modality (Figure 2).

Auditory targets were repeated tone patterns while visual targets were repetitions of faces or words within the same stimulus category. Different blocks were presented according to a randomized factorial design with visual and auditory factors exhaustively crossed in each experiment and with the additional constraint that a UV condition occurred on every 4^th^ block.

### Behavior

Hit rates to auditory targets averaged 62% with subjects correctly rejecting 97.3% of non-target tone patterns and mean reaction times (RTs) averaged 953 ms. There was no significant difference in accuracy on visual and auditory tasks (F**_(1,8)_** = 2.67, p>0.15). However, visual RTs were significantly faster than auditory RTs (672 vs. 953 ms, F**_(1,8)_** = 531.11 p<0.0001). These RT differences were due in large part to the fact that the information needed to detect visual targets was available at stimulus onset but the information needed to detect auditory targets was delayed until the third tone in the pattern had been presented (500–750 ms). Neither hit rate nor RTs were significantly influenced by Tone Frequency (F**_(2,16)_** = 0.87 and 0.49, respectively). However, auditory targets were detected more accurately in blocks with high-intensity sounds (F**_(1,8)_**  = 16.09, p<0.005). In addition, there was a Tone Frequency×Intensity interaction in both hit rate (F**_(2,16)_** = 8.48, p<0.005) and RT (F**_(2,16)_** = 4.74, p<0.05) due to greater intensity-related improvements for low- and mid-frequency tones compared with high-frequency tones. This accuracy effect was more pronounced during continuous imaging, producing a three-way interaction (Image Acquisition×Tone Frequency×Intensity, F**_(2,16)_** = 11.82, p<0.003). Neither hit rate nor RTs were influenced by the presence of visual distractors (F**_(1,8)_** = 0.10 and 0.21) or ear of delivery (F**_(2,16)_** = 2.56 and 1.29). However, auditory RTs were faster during sparse than continuous imaging conditions (−50 ms, F**_(1,8)_** = 18.42, p<0.003) and tended to be more accurate (+3.3%, F**_(1,8)_** = 5.25, p<0.06). False alarm rates averaged 2.7%. Subjects produced more false alarms during bimodal than unimodal auditory-attention conditions (+0.17%, F**_(1,8)_** = 12.31, p<0.01). No other main effects or interactions reached significance.

**Figure 3:**
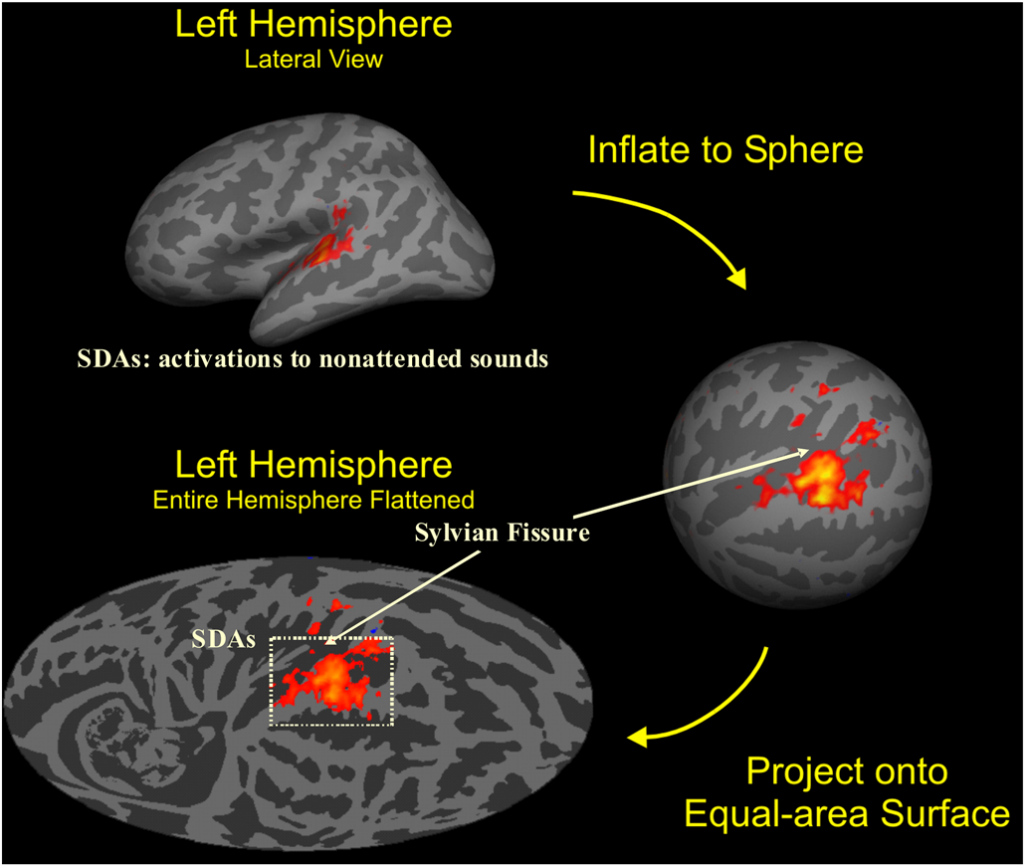
Cortical surface analysis. The cortex from each subject was segmented with FreeSurfer (Fischl, Sereno, Tootell and Dale, 1999) to show gyri (light) and sulci (dark) on the cortical surface, then inflated to a sphere and aligned to a common coordinate system The functional and anatomical data were then mapped onto a Mollweide equal-area projection after rotating the sphere so that the intersection of Heschl's gyrus (HG) and the superior temporal gyrus (STG) lay at the map center with the STG aligned along the equator. Stimulus-dependent activations (SDAs) averaged over all auditory stimulation conditions and subjects during sparse imaging experiments are shown on the average anatomy of the left hemisphere. Activations were restricted to the regions of auditory cortex near HG with the outlined region enlarged in the figures shown below. Colored voxels show significant activations (t>3.0) with mean percent signal changes ranging from 0.1–1.0% (red to yellow).

We quantified activations on the surface of auditory cortex after inflating and aligning the cortex shown in Figure 3. Across-subject analysis was performed after the data from each subject had been coregistered with a hemispherically unified spherical coordinate system obtained by aligning the reflected cortical surface of the right hemisphere with that of the left hemisphere after numerically minimizing average interhemispheric differences in cortical surface curvature using rigid body transformation of the right hemisphere.

**Figure 4:**
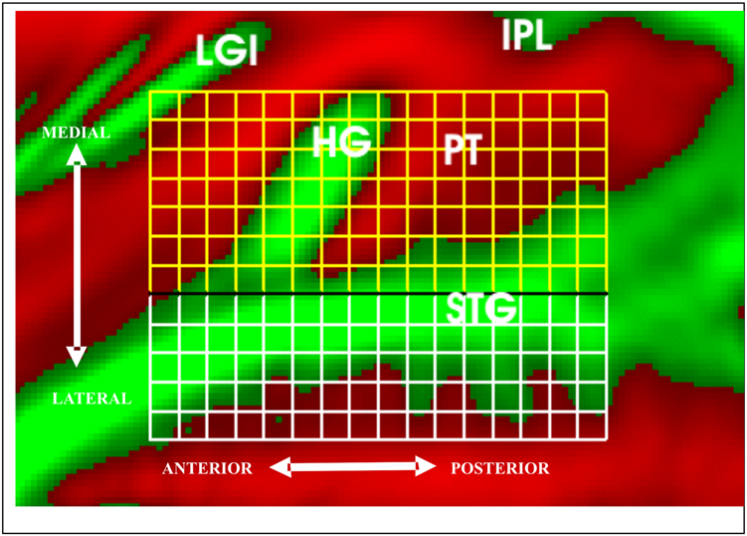
Grid measurement. Activations in auditory cortex were quantified using medial (yellow) and lateral (white) grids. Each grid contained individual grid elements of identical size (approximately 5×5 mm) on the inflated cortical surface. Average cortical surface curvature is shown (gyri = green, sulci = red). LGI = long gyri of the insula; HG = Heschl's Gyrus, IPL = inferior parietal lobe, PT = Planum Temporale, STG = superior temporal gyrus. Approximate anterior-posterior and medial-lateral directions on the inflated surface are shown.

The data were quantified in adjacent medial and lateral grids that covered auditory cortex and the adjacent superior temporal gyrus (Figure 4). SDAs and ARMs were analyzed in each grid with a 9-way ANOVA for repeated measures incorporating the following factors: subjects (treated as a random factor), tone frequency, tone intensity, ear of delivery, sparse vs. continuous imaging, hemisphere, type and/or presence of concurrent visual stimuli, and anterior-posterior (A-P) and medial-lateral (M-L) location on the grid. All-ASAs were analyzed with a 10-way ANOVA that included auditory stimulation conditions (BA, BV, and UA) as an additional attention factor along with the factors included in SDA and ARM analyses. Main effects and first order interactions were evaluated at the p<0.05 level with third- and higher-order interactions evaluated using a stricter p<0.01 criterion. F-ratios and probabilities are reported for significant results and results approaching significance, whereas F-ratios alone are presented for other comparisons. Preliminary analysis showed that neither SDAs nor ARMs changed significantly across the successive experiment sessions (F**_(2,16)_** = 1.82 and 0.49, respectively). Therefore, data were pooled across experimental sessions during the analysis of both sparse and continuous data.

### Sensory responses in medial auditory cortex

The medial grid contained large SDAs (mean 0.25% signal change, peak 0.70%) along with small ARMs (mean 0.08%, peak 0.25%). An analysis of the effects of stimulus factors produced similar results for SDA and All-ASA analyses. In the interest of brevity, All-ASA analyses are presented below with parallel SDA analyses included in supplemental materials (Table S1) unless specifically indicated.

#### Tonotopic organization

**Figure 5:**
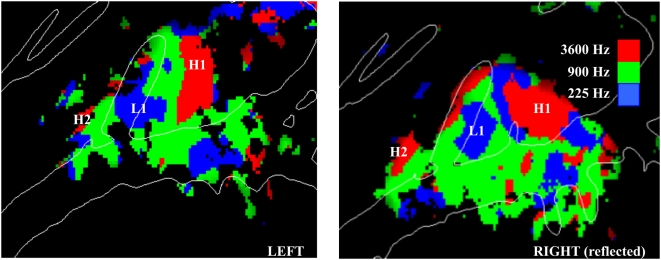
Mirror-image tonotopic organization of auditory cortex shown for the left and right (reflected) hemispheres. Plots show regions with significant activations coded by the frequency that produced maximal activation at that point. Red = 3600 Hz, Green = 900 Hz, Blue = 225 Hz. Averaged over subjects, continuous and sparse sampling, sound spatial position, and sound intensity. Activation maps are shown on the average gyral structure (white lines) of the left and right hemispheres. z-score threshold = 4.0. H1 and H2 identify posterior and anterior foci driven by high-frequency tones whereas L1 shows a central region driven by low-frequency tones.

Tonotopic z-score maps across conditions are shown for the left and right hemispheres in Figure 5. Three reliable frequency-specific regions could be identified: a high-frequency H1 region posterior to HG, a low-frequency L1 region on mid-lateral HG, and a high-frequency H2 region anterior to the intersection of anterior faces of HG and the STG. Tone Frequency systematically changed the distribution of activations in A-P (F**_(30,240)_** = 3.58, p<0.01) and M-L (F**_(12,96)_** = 5.08, p<0.01) dimensions. There were no significant interactions of these effects with Attention (F**_(60,480)_** = 1.22 for A-P, F**_(24,192)_** = 1.46 for M-L), Image Acquisition (F**_(30,240)_** = 0.98 for A-P, F**_(12,96)_** = 0.83 for M-L), or Intensity (F**_(30,240)_** = 1.18 for A-P, F**_(12,96)_** = 1.78, p>0.10 for M-L).

There was a main effect of Tone Frequency on All-ASA activation magnitudes (F**_(2,16)_** = 4.61, p<0.04): 900 Hz tones produced greater mean percent signal change (SDAs = 0.34%) than either 3600 Hz (0.28%) or 225 Hz (0.30%) tones. Therefore, frequency-related changes in distribution were further analyzed after the data had been normalized to eliminate the main effect of Tone Frequency. Following normalization, frequency-related changes persisted in A-P (F**_(30,240)_** = 3.62, p<0.01) and M-L (F**_(12,96)_** = 3.12, p<0.03) distributions. Tonotopic differences in normalized amplitudes did not differ between Hemispheres (F**_(30,240)_** = 1.39 for A-P, F**_(12,96)_** = 1.14 for M-L) or for Continuous and Sparse imaging conditions (F**_(30,240)_** = 0.96 for A-P, F**_(12,96)_** = 0.79 for M-L). There were no significant changes in normalized tonotopic distributions with intensity along A-P (F**_(30,240)_** = 1.13) or M-L (F**_(12,96)_** = 1.99, p<0.11) dimensions.

**Figure 6:**
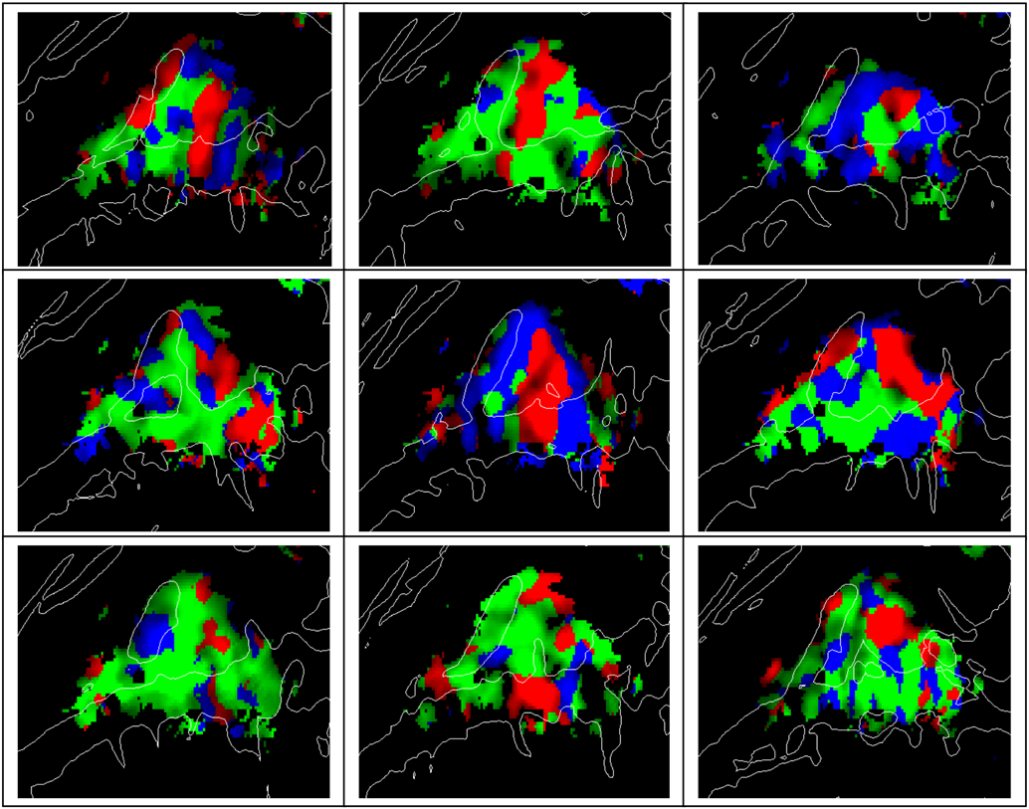
Tonotopic organization of auditory cortex in each of the nine individual subjects. Plots show regions with significant auditory activations coded by the frequency that produced maximal activation at that point. Red = 3600 Hz, Green = 900 Hz, Blue = 225 Hz. In each subject, activations were averaged over hemispheres, over continuous and sparse sampling, attention conditions, sound spatial position, and sound intensities and projected on the individual's left hemisphere cortical surface anatomy. Top: subjects 1–3, center: subjects 4–6, bottom: subjects 7–9. z-score threshold = 3.0.

The tonotopic distributions for individual subjects are shown in Figure 6. The general H1-L1-H2 pattern could be recognized in all subjects, although the relative sizes and locations of the different zones showed considerable intersubject variability. For example, the distance between HG and the H1 field varied considerably in different subjects (cf. subject 2 and subject 3) and the L1 field varied considerably in extent (cf. subject 5 and subject 8).

**Figure 7:**
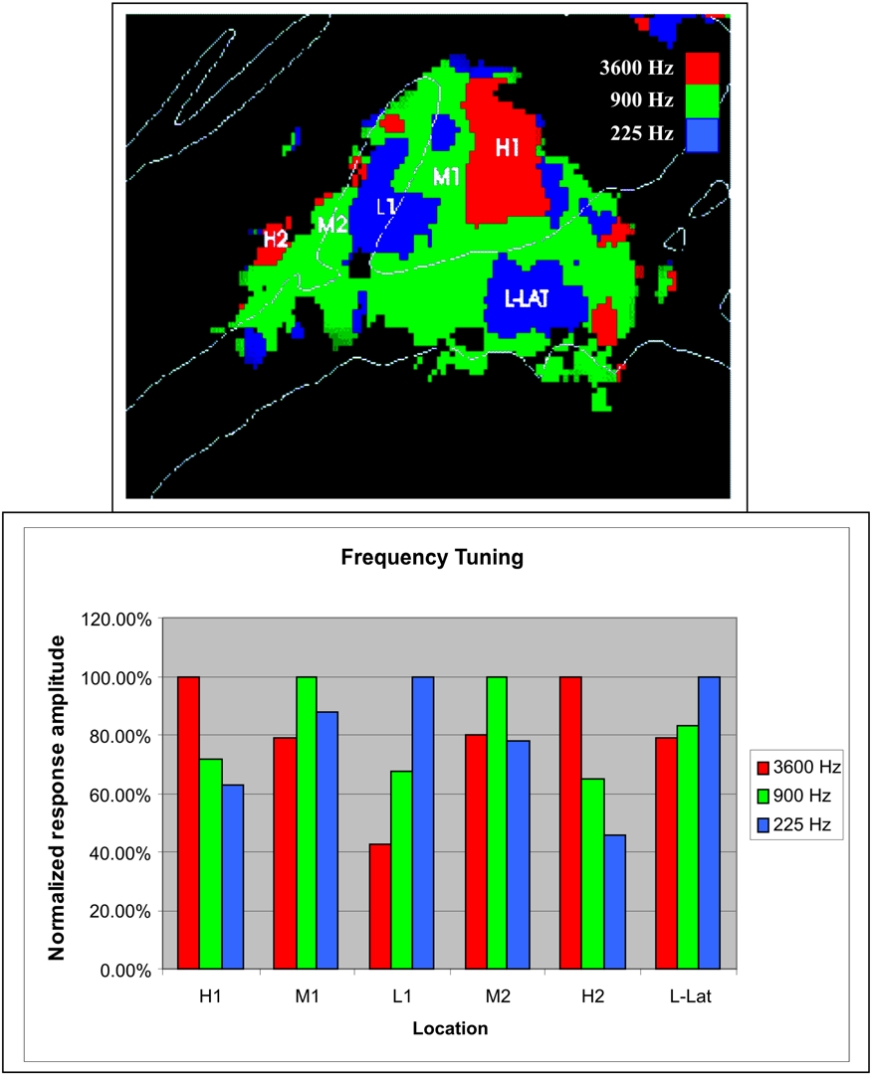
Frequency preferences at six different locations in auditory cortex. Locations of areas sampled in frequency specific regions are shown on a mean tonotopic map (top, averaged over all conditions). High- and low-frequency tuned tonotopic areas (H1, H2, and L1) showed relatively sharp tuning while regions tuned to mid-frequencies tended to also be activated to some extent by low and high frequency tones. Apparent tonotopic regions in lateral auditory cortex (L-Lat) were not sharply tuned. Normalized response amplitude shows the magnitude of response to each frequency as percentage of the response to the preferred frequency.

The nature of the tonotopic gradients was further clarified by examining frequency tuning in the population-averaged data (Figure 7). Zones H1 and H2 were selective for 3600 Hz tones, with smaller responses for 900 Hz tones and a further reduction for 225 Hz tones. The low-frequency L1 zone showed an opposite pattern. Sites intermediate between high-and low-frequency zones showed maximal activations for intermediate frequency tones (900 Hz). Thus, in medial auditory areas there were tonotopic gradients connecting high- and low-frequency zones and intermediate regions that were tuned to intermediate frequencies. In contrast, an apparent low-frequency specific zone in lateral regions (L-Lat) showed less frequency specificity.

#### Inter-hemispheric differences

All-ASA activations did not differ in magnitude in the left and right hemispheres (F**_(1,8)_** = 2.61, p<0.15) and there were no significant interactions between Hemisphere and Tone Frequency (F**_(2,16)_** = 0.54), Tone Intensity (F**_(1,8)_** = 0.01), or Image Acquisition (F**_(1,8)_** = 0.10). Moreover, Tone Frequency×Hemisphere×A-P (F**_(30,240)_** = 1.11) and Tone Frequency×Hemisphere×M-L (F**_(12,96)_** = 1.02) interactions were also absent, suggesting similar patterns of tonotopic organization in the two hemispheres.

#### Effects of unattended visual stimuli

The effects of unattended visual stimuli on activations in medial auditory cortex were analyzed by comparing activation magnitudes in bimodal and unimodal auditory-attention conditions (UA vs. BA). The magnitude of auditory activations was slightly smaller when unattended visual stimuli were presented (BA vs. UA, −0.02%), but this effect failed to reach statistical significance (F**_(1,8)_** = 3.84, p<0.09).

#### Tone intensity

**Figure 8:**
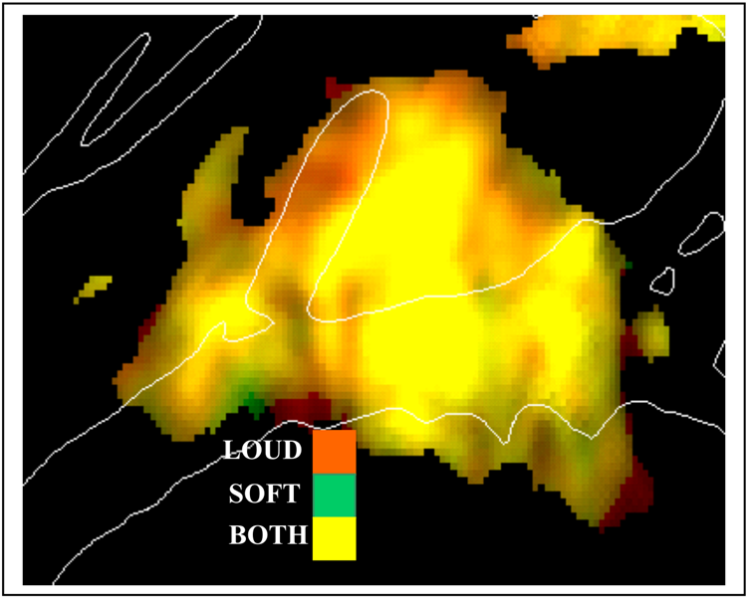
Effects of tone intensity. Significant activations (z-score range 5.0–20.0) are shown with the degree of saturation reflecting the magnitude of z-score, and the color showing relative magnitude of activation for different sound intensities: Red = intense, green = soft yellow = both. Data were averaged over subjects, spatial positions, attention, frequencies, image acquisition, and hemispheres.

Figure 8 shows overlaid activation maps to loud (90 dB) and soft (70 dB) sounds averaged across sparse and continuous imaging conditions and hemispheres. The main effect of Intensity was significant (F**_(1,8)_** = 8.54, p<0.02) but it did not interact with Tone Frequency (F**_(2,16)_** = 2.67, p<0.11), Image Acquisition (F**_(1,8)_** = 0.61), Ear of Delivery (F**_(2,16)_** = 1.69), Attention (F**_(1,8)_** = 0.05) or Hemisphere (F**_(1,8)_** = 0.01). However, there was a significant intensity-related change in A-P distribution (F**_(15,120)_** = 6.38, p<0.001) and a trend toward shifts in M-L distribution (F**_(6,48)_** = 2.77, p<0.07). Following amplitude normalization to eliminate the main effect of intensity, both A-P (F**_(15,120)_** = 4.17, p<0.01) and M-L shifts (F**_(6,48)_** = 5.75, p<0.003) became significant: louder tones produced relatively greater activations medially and anteriorly. The intensity-related shift in distribution was similar in continuous and sparse imaging conditions (F**_(6,48)_** = 0.40 and F**_(15,120)_** = 0.69) and for tones of different frequencies (F**_(12,96)_** = 1.40 and F**_(30,240)_** = 0.98).

#### Ear of delivery

There were no significant main effects of Ear of Delivery (F**_(2,16)_** = 2.41). However, there was a highly significant Ear of Delivery×Hemisphere interaction (F**_(2,16)_** = 13.03, p<0.0005). This was due primarily to the fact that monaural sounds produced larger activations in the hemisphere contralateral to stimulation (0.33%) than in the ipsilateral hemisphere (0.25%) whereas binaural sounds produced activation magnitudes in both hemispheres (0.34%) that were similar to those produced by contralateral monaural sounds.

When binaural blocks were excluded from the analysis, the Ear of Delivery×Hemisphere interaction persisted (F**_(1,8)_** = 14.81, p<0.0003). There was also a significant difference in distribution of activations over the ipsilateral and contralateral hemispheres in the A-P direction (F**_(15,120)_** = 5.21, p<0.0001). This effect persisted after normalization to equate ipsilateral and contralateral activation magnitudes (F**_(15,120)_** = 3.70, p<0.01) and a significant effect was also observed in M-L distribution (F**_(6,48)_** = 3.50, p<0.03). These changes reflected the fact that, in comparison with the distribution of activations produced by ipsilateral tones, the distribution of activations produced by contralateral tones was disproportionately enhanced at anterior and medial grid locations.

**Figure 9:**
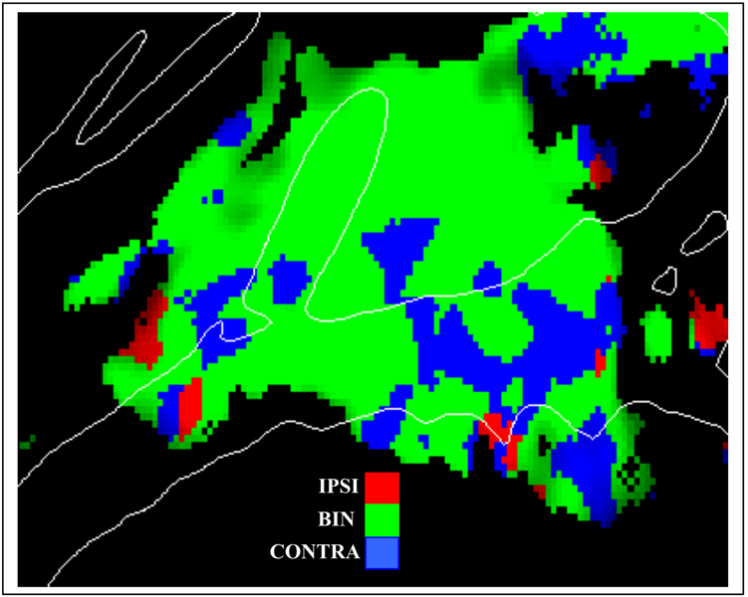
Effects of Ear of Delivery. Significant activations with the color showing the tone location producing maximal activation at that point on the cortical surface. Red = ipsilateral ear, green = binaural, blue = contralateral ear. Averaged over subjects, attention, image acquisition, frequencies and hemispheres. z-score threshold = 4.0.

Figure 9 shows the effects of the relative spatial position of tones at each point on the cortical surface, demonstrating that binaural and contralateral tones produced more widespread activations than ipsilateral tones, but that many regions responded to tones in all spatial positions. Although binaural tones produced slightly enhanced amplitudes over most of the medial auditory cortex in comparison with contralateral tones, this difference failed to reach statistical significance (F**_(1,8)_** = 0.19). In addition, there were no significant differences in the distribution of activations produced by contralateral and binaural sounds in either A-P (F**_(15,120)_** = 1.62) or M-L (F**_(6,48)_** = 0.41) directions. Furthermore, there were no significant interactions between contralateral vs. binaural tone locations and Tone Frequency (F**_(1,8)_** = 1.74), Attention (F**_(2,16)_** = 0.47), Image Acquisition (F**_(1,8)_** = 1.90), Intensity (F**_(1,8)_** = 1.16), or Hemisphere (F**_(1,8)_** = 1.03). Thus, in medial regions of auditory cortex the distributions and functional properties of activations produced by binaural sounds were largely indistinguishable from those produced by contralateral sounds.

#### Image acquisition

**Figure 10:**
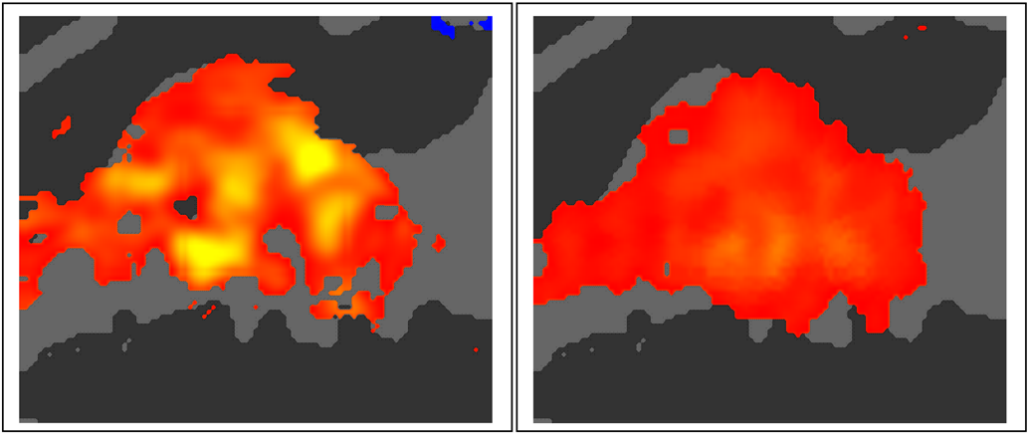
Effects of image acquisition parameters. Stimulus dependent activations in sparse (left) and continuous (right) sampling conditions averaged over tone parameters, hemispheres and subjects. Scale ranges from 0.1% (red) to 1.0% (yellow) combined with a z>3.0 mask.

Figure 10 shows mean activations during sparse and continuous imaging conditions. All-ASAs were larger with sparse (mean 0.38%) than continuous (0.23%) Image Acquisitions (F**_(1,8)_** = 23.75, p<0.002). There were no significant interactions between the Image Acquisition factor and any other stimulus or task factor including Frequency (F**_(2,16)_** = 0.35), Ear of Delivery (F**_(2,16)_** = 1.25), Intensity (F**_(2,16)_** = 0.61) or Attention (F**_(2,16)_** = 2.55, p>0.10), nor did Image Acquisition differentially affect the two hemispheres (F**_(1,8)_** = 0.10). However, there were significant differences in the Anterior-Posterior (A-P) distributions during sparse and continuous Image Acquisitions (F**_(15,120)_** = 2.83, p<0.04). After data normalization to eliminate the main effect of the Image Acquisition factor, A-P differences were reduced (F**_(15,120)_** = 1.79, p<0.20), but highly significant differences in M-L distribution emerged (F**_(6,48)_** = 16.35, p<0.0001). This reflected relatively larger amplitudes at mesial grid locations during sparse imaging.

### Attention effects in medial auditory cortex

**Figure 11:**
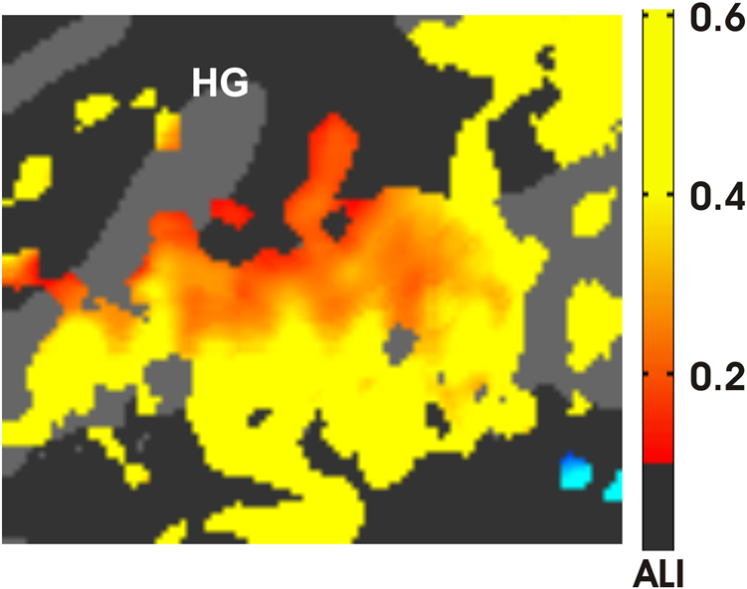
Effects of attention. The attentional lability index (ALI) for areas showing significant attention effects. Data were averaged over sound parameters, hemispheres, subjects, and image acquisition protocols. Activations in blue regions in the STS (lower right) were enhanced during visual attention. HG = Heschl's Gyrus. z-score threshold = 4.0.

There were a significant differences in activation magnitudes in medial auditory regions in different auditory attention conditions (F**_(2,16)_** = 43.61, p<0.0001): activations were larger during the two auditory attention conditions (bimodal and unimodal, mean 0.32% and 0.34%) than during visual attention (0.25%). We estimated the relative magnitude of attentional effects vs. sensory responses using an attentional lability index (ALI), defined as the ARM amplitude divided by the summed amplitude of ARMs + SDAs. Thus, the ALI could range from 0.0 (for no attentional enhancement) to 1.0 (for regions activated exclusively by attended stimuli). The ALI measure over the medial grid averaged 0.24. Figure 11 shows the distribution of ALIs over those regions that showed significant attentional modulation. Significant attentional modulation was seen in lateral regions of tonotopic fields H1, L1, and H2 as well as anterior to HG. ALI magnitudes increased further in the lateral grid (see below).

The characteristics of attentional modulation were further analyzed with ARM ANOVAs. ARM magnitudes were not affected by Tone Frequency (F**_(2,16)_** = 0.60), Intensity F**_(1,8)_** = 0.02), Ear of Delivery (F**_(2,16)_** = 0.15), or Image Acquisition (F**_(1,8)_** = 4.32, p<0.08), Type of Visual distractor (F**_(1,8)_** = 0.35) or Hemisphere (F**_(1,8)_** = 0.34). ARM magnitudes varied in the M-L dimension (F**_(6,48)_** = 3.63, p<0.05, being largest in most lateral locations in the medial grid), but not across the A-P dimension (F**_(15,120)_** = 2.26, p<0.11). ARMs showed a trend toward an opposite Ear of Delivery×Hemisphere interaction from that seen for sensory responses: ARMs tended to be larger over the hemisphere ipsilateral to the stimulated ear than over the contralateral hemisphere (F**_(2,16)_** = 4.00, p<0.09). Other second-order interactions did not reach significance. In particular, there was no evidence of tonotopic changes in ARM distributions (e.g., Frequency×A-P F**_(20,160)_** = 1.21, Frequency×M-L F**_(12,96)_** = 0.37). Nor did ARM distributions change with sound Intensity (M-L, F**_(6,48)_** = 0.94, A-P F**_(15,120)_** = 1.35) or Image Acquisition (M-L F**_(6,48)_** = 1.66, A-P F**_(15,120)_** = 0.81).

**Figure 12:**
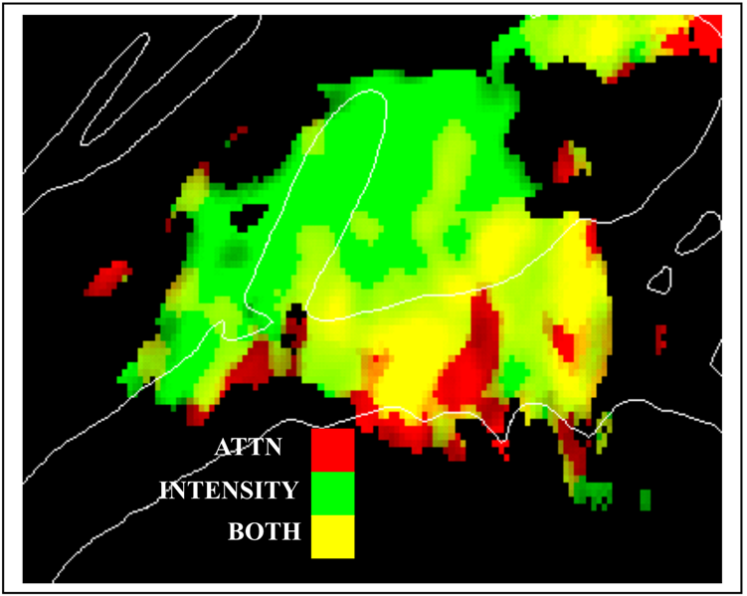
Effects of intensity vs. attention. Significant activations over a z-score range of 5.0 to 10.0 with the degree of saturation showing the z-score, and the color showing relative magnitude of activation for attention effects (Red) and Loud-Soft differences in activation magnitude (Green). Yellow = both attention and intensity. Data averaged over subjects, locations, image acquisition, frequencies and hemispheres.

Figure 12 shows the effects of attention compared with the effects of increasing stimulus intensity. These two manipulations had markedly different effects on auditory activations. Increasing tone intensity resulted in increased activations throughout medial auditory cortex whereas attention enhanced activations primarily in lateral regions of auditory cortex along the STG and in mesial regions anterior to HG.

#### Distributions of ARMs and SDAs

A comparison of normalized distributions of ARMs and SDAs in the medial grid showed a significant difference in the A-P dimension (F**_(15,120)_** = 4.47, p<0.006) due to the fact that ARMs were more posterior than SDAs. No ARM/SDA differences were seen in the M-L dimension (F**_(6,48)_** = 0.70). There was also a significant Condition×Localization×Hemisphere interaction (F**_(2,16)_** = 6.95, p<0.02) due primarily to the fact that SDAs were enhanced contralaterally to the stimulated ear whereas ARMs tended to be enhanced ipsilaterally.

#### Amplitude changes of sensory responses and attention effects within blocks

**Figure 13:**
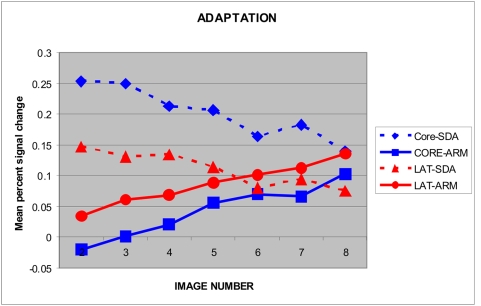
Adaptation of SDAs and ARMs. Mean percent signal change for SDAs and ARMs in medial and lateral grids across successive images 2–8 of each block during continuous sampling.

Amplitude changes in SDAs and ARMs were examined over images 2–8 in continuous imaging blocks (Figure 13). SDAs were largest at the beginning of stimulus blocks and significantly declined throughout blocks (F**_(6,48)_** = 4.60, p<0.02). This SDA adaptation effect showed no significant interactions with any other factor. ARMs showed a pattern that was opposite to that of SDAs: ARMs increased in amplitude over the block (F**_(6,48)_** = 4.65, p<0.02).

### Sensory responses in lateral auditory cortex

In the lateral grid SDA amplitudes were reduced in comparison with those in the medial grid (mean 0.12%, peak 0.54%), while ARM amplitudes were increased (mean 0.10%, peak 0.25%). This produced an increase in the ALI (0.45) in comparison with the medial grid (Figure 11). SDAs in the lateral grid were largest in amplitude along the medial STG and declined steeply at more lateral locations in the superior temporal sulcus (STS,F**_(4,32)_** = 37.58, p<0.0001). SDA magnitudes did not vary significantly with A-P position (F**_(15,120)_** = 2.41, p<0.07). As in the medial grid, the effects of acoustic features on sensory activations in the lateral grid were virtually identical in All-ASA analyses (presented below) and SDA analyses (included in supplemental materials, Table S2).

#### Tone frequency

Unlike activations in the medial grid, activations in the lateral grid showed no main effect of Tone Frequency (F**_(2,16)_** = 0.50) nor were there significant interactions between Tone Frequency and A-P grid location (F**_(30,240)_** = 0.95). However, there was a borderline Tone Frequency×M-L interaction (F**_(8,64)_** = 3.03, p<0.05) that primarily reflected relatively increased relative activation magnitudes to mid-frequency tones at mesial locations in the lateral grid.

#### Tone intensity

Tone intensity did not significantly affect All-ASA magnitudes in the lateral grid (F**_(1,8)_** = 0.04), nor did it significantly alter activation distributions (A-P, F**_(15,120)_** = 0.84; M-L (F**_(4,32)_** = 1.17).

#### Image acquisition

Unlike sensory activations in the medial grid, All-ASAs in the lateral grid were not significantly influenced by Image Acquisition (F**_(1,8)_** = 1.19), nor did Image Acquisition significantly alter A-P (F**_(15,120)_** = 0.55) or M-L (F**_(4,32)_** = 2.83, p<0.10) distributions.

#### Hemispheric differences

In contrast to the results seen in the medial grid, there was a main effect of Hemisphere in the lateral grid (F**_(1,8)_** = 7.94, p<0.03): All-ASAs were larger over the right (0.24%) than left (0.14%) hemisphere. Following amplitude normalization to eliminate the main effect of hemisphere, there were no interhemispheric differences in distributions in either the A-P (F**_(15,120)_** = 0.94) or M-L dimensions (F**_(4,32)_** = 0.94).

#### Ear of delivery

There was an Ear of Delivery×Hemisphere interaction (F**_(2,16)_**  = 12.96, p<0.0005). This was due to larger right hemisphere All-ASA amplitudes for tones presented binaurally (right hemisphere = 0.27%, left hemisphere = 0.13%) than for tones presented to the left (0.25% vs. 0.13%) or right ear (0.21% vs. 0.17%). When activations to binaural sounds were excluded from the analysis, activations were found to be larger in the hemisphere contralateral to the ear of stimulation (F**_(1,8)_** = 8.56, p<0.02). As in the medial grid, there were no significant overall differences in the amplitude of activations to binaural vs. contralateral sounds (F**_(1,8)_** = 0.11). Nor were there interactions between contralateral vs. binaural activations and Attention (F**_(2,16)_** = 1.49), Image Acquisition (F**_(1,8)_** = 2.08), Tone Frequency (F**_(1,8)_** = 1.25), or Intensity (F**_(1,8)_** = 0.40). The distributions of All-ASAs following contralateral and binaural tones did not differ significantly in A-P (F**_(15,120)_** = 0.66) or M-L (F**_(6,48)_** = 0.16) dimensions. Nor were there significant interactions between Sound Location (contralateral vs. binaural) and Hemisphere (F**_(1,8)_** = 3.20, p<0.12). Thus, the distributions and functional properties of activations in the lateral grid produced by binaural sounds were similar to those produced by contralateral sounds.

#### Effects of unattended visual stimuli on lateral auditory cortex

There was no significant effect of unattended visual stimuli on activations in the lateral grid (F**_(1,8)_** = 0.14), but there was a significant interaction with the type of visual stimulus (words vs. faces, F**_(1,8)_** = 5.83, p<0.05). This reflected a small increase in lateral activations when word stimuli were presented (+0.03%) and a slight reduction (−0.02%) when face stimuli were presented. This effect showed no interaction with Hemisphere (F**_(1,8)_** = 0.27) or any other factor.

### Attention effects in lateral auditory cortex

Activations were enhanced by attention, producing a highly significant main effect of condition (F**_(2,16)_** = 16.49, p<0.002): attended sounds produced considerably larger responses (UA = 0.23%, BA = 0.22%) than unattended sounds (BV = 0.13%). As shown in Figure 11, significant ARMs with high ALIs covered the STG and declined in amplitude in the STS (F**_(4,32)_** = 6.49, p<0.02). ARM magnitudes also varied with A-P position (F**_(15,120)_** = 3.87, p<0.006) with maximal amplitudes in posterior grid locations.

As in the medial grid, ARMs in the lateral grid were not significantly influenced by Tone Frequency (F**_(2,16)_** = 0.46), Intensity (F**_(1,8)_** = 0.00), Ear of Delivery (F**_(2,16)_** = 2.50, p<0.15), or Type of Visual Distractor (F**_(1,8)_** = 2.12), nor were they significantly influenced by Image Acquisition (F**_(1,8)_** = 1.46). Attention effects were similar in magnitude over the two hemispheres (F**_(1,8)_** = 0.56). There were no significant interactions between hemisphere and ARM distribution (A-P (F**_(15,120)_** = 0.74; M-L F**_(4,32)_** = 2.31), nor was their evidence of tonotopic organization (e.g., Tone Frequency×A-P position, F**_(30,180)_** = 1.03; Tone Frequency×M-L position F**_(8,64)_** = 1.53). The only other significant finding was an unexpected interaction between Image Acquisition and Ear of Delivery (F**_(2,16)_** = 4.35, p<0.04) which was due to larger ARMs following right-ear stimulation during continuous image acquisition and larger ARMs following left-ear stimulation during sparse image acquisition.

#### Amplitude changes of sensory responses and attention effects within blocks

As in the medial grid, SDA amplitudes in the lateral grid tended to decline over the block, but the results failed to reach significance (F**_(4,40)_** = 2.44, p<0.11). In contrast, ARMs increased in amplitude over the block (F**_(6,48)_** = 4.20, p<0.02).

#### Distributions of ARMs and SDAs

A comparison of normalized distributions of ARMs and SDAs in the lateral grid showed a significant difference in M-L distribution (F**_(4,32)_** = 13.58, p<0.001) due to the fact that ARMs were more laterally distributed than SDAs. There was also a significant Activation-Type×Hemisphere interaction (F**_(1,8)_** = 6.14, p<0.05) reflecting the fact that SDAs in lateral regions were larger over the right hemisphere whereas ARMs were symmetrically distributed.

## Discussion

### Independent processing of auditory features

The factorial design of the current experiment enabled us to analyze the main effects and interactions of a number of important acoustic features including tone frequency, intensity, ear of delivery, and imaging acquisition parameters. While each of these factors significantly influenced the magnitude and distribution of activations in medial auditory cortex, there were negligible interactions between them. These results suggest that the processing of different acoustic features in auditory cortex occurs independently and in parallel.

### Functional organization of medial regions of auditory cortex

We found evidence of reliable mirror-symmetric tonotopic organization in medial auditory cortex that was similar to that previously reported in individual subjects by Formisano and colleagues [2]. Specifically, we found a large H1 region medial and posterior to HG that was tuned to high frequencies, an L1 region located on mid-lateral HG that was tuned to low-frequencies, and a third, smaller H2 region anterior to HG that was tuned to high frequencies (Figure 7). Intermediate regions were tuned to mid-frequencies. As in Formisano et al [2] these distributions most likely activity in two mirror-symmetric tonotopic fields: A1 (connecting H1 and L1) and R (connecting L1 and H2). The relatively smaller apparent extent of the anterior H2 region may relate to recent findings that anterior fields R and RT are less responsive to pure tones than A1 and may also be tuned to lower sound intensities [54].

The mirror-symmetric tonotopic pattern seen in the current experiment is similar to the tonotopic organization seen in fMRI studies of macaque auditory cortex [13]. As in the macaque, the frequency-specific regions that we observed in human auditory cortex had relatively large dimensions and spanned most of medial auditory cortex. The high-frequency region H1 (Figure 7) had a medial-lateral extent of approximately 25 mm, which is slightly more than twice the length of the isofrequency contour seen in the posterior high-frequency region of the macaque. In the macaque, this high-frequency region is hypothesized to include the core field A1 as well as belt fields CL and MM [13]. The approximate two-fold increase in the size of core fields of human cortex with respect to comparable fields in the macaque [55] suggests that the frequency-specific activations observed in the current study similarly include contributions from both core and belt fields.

Although all subjects showed the general tonotopic pattern with H1, L1 and H2 regions, the precise location and extent of these regions varied substantially in different subjects. Population averaging isolated regions where frequency tuning was similar across subjects. While additional frequency-specific regions may be apparent in single subject analyses [11], [12], their locations were not sufficiently consistent to survive across-subject averaging in the current experiment. The spatial smearing inherent in across-subject averaging would also increase the blurring of adjacent core and belt areas with similar frequency tuning in average data and obscure the contributions of smaller tonotopic fields with greater variation in anatomical location.

**Figure 14:**
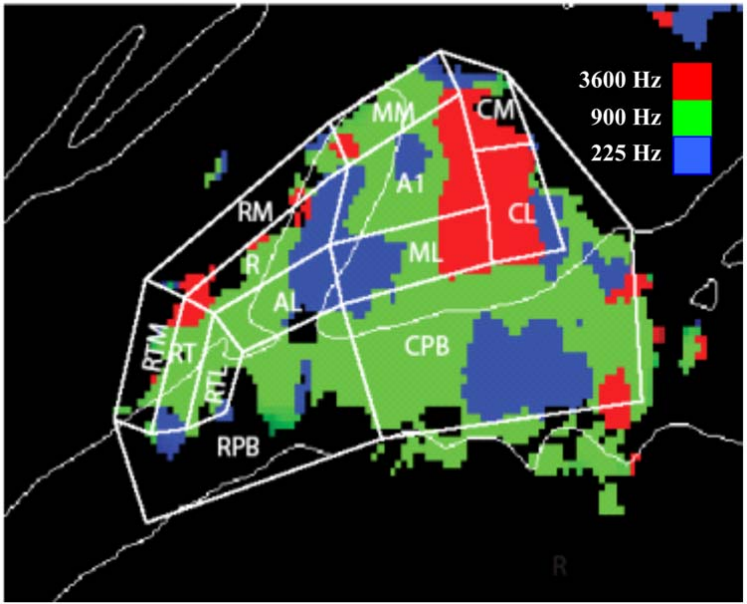
A schematic model of human auditory cortical fields. Schematic representations of primate auditory cortical fields (Kaas & Hackett, 2000) superimposed on frequency-specific activation patterns in human auditory cortex. Field borders were estimated based on similarities in tonotopic organization observed in the current study and in fMRI studies of macaque auditory cortex (Petkov et al. 2006, Kayser et al. 2007). Colors show frequency tuning of grand mean activations averaged over subjects, hemispheres, image acquisition parameters, tone location and tone intensity. Red = 3600 Hz, green = 900 Hz, blue = 225 Hz. A1 = primary auditory cortex; R = rostral, T = temporal, M = middle; A = Anterior; L = lateral, C = caudal, PB = parabelt.

Anatomical and functional studies of auditory cortex in the macaque have revealed thirteen different ACFs [56]. Figure 14 shows a schematic model of these fields superimposed on the grand mean tonotopic maps from the current study using a model similar to models of macaque auditory cortex [13], [57]. The model assumes that frequency-selective activations occur primarily at borders between ACFs that share common frequency tuning. For example, the H1 region is hypothesized to reflect combined activations in the high-frequency region of A1 as well as high-frequency regions in four surrounding mirror-symmetric belt fields. Similarly, L1 would reflect activations in the anterior region of A1 that is responsive to low frequencies as well as activations in surrounding low-frequency regions in R and lateral belt fields ML and AL. Thus, the principal tonotopic axis connecting fields H1-L1-H2 would include activations in core and belt regions. However, in comparison with existing maps of macaque auditory cortex, activations in human auditory cortex reveal more extensive activations in non-tonotopic regions that are lateral and posterior to the tonotopic representations. These non-tonotopic activations likely arise in caudal regions of auditory cortex that are equivalent to parabelt fields and that include Tpt [58], a region that may have undergone expansion in humans relative to other primate species [59].

While previous studies that have variously failed to find tonotopic organization in the right [7], [10], [15] or left [16], [17] hemispheres, our study suggests that both hemispheres contain similar mirror-symmetric tonotopic maps. Moreover, the tonotopic maps in the left and right hemispheres had similar locations relative to surrounding anatomical landmarks. Finally, we found no influence of sound intensity on tonotopic map organization. While only limited sound intensities were studied, this result is consistent with recent non-human primate findings showing that the frequency tuning is well-preserved across a range of sound intensities [60].

Our results, like those of Formisano [2] suggest that functionally-defined core auditory fields run obliquely to the principal anatomical axis of HG. In contrast, anatomical studies of human auditory cortex report that core areas run along HG or at very shallow oblique angles [55], [61], [62]. One possible explanation for this discrepancy is that fMRI BOLD signals may have been displaced from the site of origin due to venous drainage into sulcal regions anterior and posterior to HG [63], [64]. Alternatively, lateral and posterior belt regions may be disproportionately activated in the human brain relative to the macaque brain, displacing the center of mass of frequency-specific zones toward belt fields in the planum temporale and the STG.

There was also a main effect of tone frequency on auditory activations in medial auditory cortex: activation magnitudes were larger to 900 Hz tone patterns than to tone patterns centered at 225 or 3600 Hz. This effect was somewhat surprising since the sound spectrum of scanner noise showed maximum amplitudes in the mid-frequency range (640 Hz) that would have been expected to mask mid-frequency tones more effectively than tones of other frequencies. One possible explanation is that the intermittent scanner noise resulted in long-term potentiation of responses near the scanner noise peaks [65]. Alternatively, scanner noise may have adapted neuronal populations that would normally have inhibited responses to the 900 Hz tones [39]. Finally, it is possible that human auditory cortex is preferentially tuned to middle frequencies (500–2000 Hz) because of their importance in conveying the phonological information in speech [66].

### Functional specialization within isofrequency bands

We found that activations increased in magnitude and extent with increases in sound intensity [16], [22], [67]. Moreover, the distribution of activations shifted medially and anteriorly in response to louder sounds. These results are consistent with those of Bilecen and colleagues [3] who reported a similar intensity-related displacement of activations. Similar shifts in activation distribution were seen during sparse vs. continuous imaging and following contralateral sounds vs. ipsilateral sounds. These medial-anterior displacements were largely orthogonal to the tonotopic axis of core and belt auditory fields consistent with the hypothesis that neuronal populations that code stimulus intensity, ear of delivery, and signal-to-noise ratio are non-randomly distributed in the isofrequency dimension orthogonal to the primary tonotopic axis [68]. Alternatively, the shifts in the distribution of activations may reflect different tuning properties for different ACFs. For example, medial ACFs may be more responsive to stimuli with higher intensities and/or higher signal-to-noise ratios than more lateral ACFs [69].

### Functional organization of lateral auditory cortex

In contrast to activations in medial auditory cortex, activations in lateral auditory cortex were uninfluenced by sound intensity, frequency, or image acquisition parameters and showed no evidence of tonotopic organization. Moreover, lateral auditory cortex showed greater attentional lability than medial regions. This difference is consistent with the hypothesis that medial regions extract basic auditory features whereas lateral belt and parabelt regions process more abstract, attentionally-relevant attributes of sounds [25], [70]. We also found evidence of complex cross-modal interactions in the lateral grid. These complex interactions also support the hypothesis that parabelt regions are engaged in higher-level integration of complex stimulus features.

### Auditory processing in the left and right hemispheres

Unlike previous studies that have variously reported larger activations to non-speech stimuli in the left [20], [71] or right [72] hemispheres, we found no interhemispheric differences in the extent or magnitude of sensory activations in medial auditory regions. Nor were there interhemispheric differences in the effects of Tone Frequency, Intensity, relative Ear of Delivery (e.g., ipsilateral vs. contralateral), or Image Acquisition. These results suggest that medial auditory regions in the left and right hemispheres have similar functional organization.

In contrast, activations in lateral auditory cortex were significantly enhanced in the right hemisphere. This result is consistent with the specialization of right hemisphere auditory cortex for the sensory analysis of nonlinguistic sounds such as pure tones [73] and tone sequences [43]. Since these differences emerged primarily in lateral regions of auditory cortex, it is tempting to speculate that corresponding regions of the left hemisphere may be specialized for the analysis of acoustic features that characterize linguistic stimuli [74].

### Functional imaging of auditory cortex with continuous vs. sparse acquisitions

We replicated the well-established finding that activation magnitudes were larger with sparse than continuous image acquisitions [34], [38], [75], [76]. However, aside from enhanced activation magnitudes and a slight anterior/medial shift in distributions, data from sparse and continuous imaging conditions produced similar functional maps. In particular, there were no interactions between image acquisition parameters and other sound properties including frequency, intensity, or ear of delivery. The similarity in functional results may reflect the fact that four times more images were acquired during continuous than sparse imaging, enhancing the image quality of continuous data [5]. Another contributing factor may have been the use of broadband 70 dB SPL masking noise in both continuous and sparse imaging conditions and the attenuation of scanner noise provided by insert earphones and ear protectors. These factors would tend to minimize the acoustic differences between sparse and continuous imaging conditions.

### The influence of non-attended visual stimuli on auditory cortex activations

A number of previous studies have found that activations in auditory cortex are reduced when sound-alone conditions are compared to conditions where sounds are presented in association with simultaneous visual stimuli [77], [78]. The reductions that we observed were smaller than those seen in these previous studies, perhaps because auditory and visual stimuli were presented asynchronously and during demanding intermodal attention conditions that may have further reduced cross-modal interactions [79]. Visual stimuli had complex influences on activations in lateral auditory areas that depended on the nature of the visual stimulus. Slight increments in activation were seen when attended sounds were presented in association with visually presented words. Visually presented words have been reported to directly activate auditory cortex in the absence of sounds [80]. Thus, in the current experiment, the occasional covert processing of the visual words might have induced activations that summed with those produced by auditory stimuli in lateral regions.

### Ear of delivery effects

As in previous studies [4], [27], [28], [31] monaural sounds produced larger activations in auditory cortex contralateral to the stimulated ear, while binaural sounds produced activations similar in magnitude and functional characteristics to those produced by contralateral monaural sounds [4]. Neurons in auditory cortex are usually excited by contralateral sounds, with different neurons inhibited, uninfluenced, or excited (E-I, E-O and E-E units, respectively [68] by ipsilateral sounds. The fact that contralateral sounds produced activations with a more medial and anterior distribution than did ipsilateral sounds suggests that the ratio of these cell types may differ within and between different ACFs (e.g., relatively more E-E units in lateral and posterior ACFs). Otherwise, ear of delivery did not interact with other acoustic features nor did it significantly influence the distribution of tonotopic maps. This lack of interaction suggests that ear of delivery affected processing in a relatively uniform manner across medial regions, consistent with hypotheses that neurons with different ear-of-delivery tuning are interdigitated at fine spatial scale in auditory fields [81]. In the lateral grid, we found enhanced responses to binaural sounds in the right hemisphere. This result is consistent with suggestions that the lateral auditory cortex of the right hemisphere is particularly sensitive to binaural cues such as those necessary to perceive sound movement [33].

### Within-block changes in sensory and attentional processing

Consistent with our previous results [28] SDAs declined rapidly within stimulus blocks whereas ARMs increased. Other fMRI studies have found evidence of sensory adaptation when sounds are repeated [82]. The differences between adaptation functions for attentional and sensory responses is consistent with observations that the relative magnitude of attentional modulation increases at higher rates of stimulus delivery [46].

However, there was no evidence that attention specifically reduced the adaptation of sensory responses [83]. Rather, it appeared to reflect the addition of ARMs that arose in different neuronal populations than SDAs. In addition, aspects of the current experimental design may have delayed ARM onset. For example, ARM onsets may have been delayed by the time required for attention to shift to the auditory modality following a change in the visual cue. Moreover, tone-pattern comparisons would have been delayed until at least one complete tone pattern had been presented.

#### Attentional modulation of auditory cortex

Attending to auditory stimuli in the intermodal selective attention task increased sound-related activations in auditory cortex [44], [45]. However, activations associated with obligatory sensory processing (SDAs) and attentional modulations (ARMs) had distinct properties. In particular, SDAs were strongly modulated by the acoustic features of stimuli (e.g., frequency, intensity, ear of delivery and image acquisition) whereas ARMs were unaffected. Moreover, SDAs and ARMs had distributions that differed both on a coarse scale (SDAs were larger in the medial grid, and ARMs larger in the lateral grid) and at finer scales within each grid.

In the current study, attention did not simply amplifying sensory activations in a manner similar to increasing sound intensity and signal-to-noise ratio. Attention enhanced activations predominantly in non-tonotopic lateral regions of auditory cortex, whereas increasing sound intensities enhanced activations in medial tonotopic regions. ARMs and SDAs also differed in functional organization. For example, SDAs showed a tonotopic organization whereas ARMs did not. Similarly, SDAs were enhanced over the hemisphere contralateral to the ear of stimulation, while ARMs tended to be larger over the ipsilateral hemisphere.

While these results reveal clear dissociations between ARMs and SDAs, the differences may reflect the particular attentional operations that were required in the current task: subjects performed a one-back matching task that stressed auditory short-term memory for tone patterns. Similar auditory recall tasks have been shown to activate lateral regions of auditory cortex [84], [85]. However, the level at which attention modulates neuronal processing may depend on the processing operations needed to discriminate attended and unattended stimuli [49]. Although we found that ARMs were larger over the hemisphere ipsilateral to stimulation, auditory spatial attention tasks have found that attention effects are enhanced over the hemisphere contralateral to the attended ear [30], [44], [86]–[88]. Thus, the regions of auditory cortex that are modulated by attention may depend on the level and type of attentional selection required. Because subjects in the current experiment were not required to discriminate relevant and irrelevant sounds based on their acoustic features, attentional modulation in the core regions encoding these features may have been minimized.

### Conclusions

Medial regions of auditory cortex showed large sensory responses with a mirror-symmetric tonotopic organization that was similar in the two hemispheres and that conformed to the general pattern seen in non-human primates. Distribution analysis suggested that both tone intensity and signal-to-noise ratio shifted activation distributions within isofrequency bands. Activations to monaural tones were enhanced over the hemisphere contralateral to stimulation while the distribution and magnitude of activations to binaural tones were indistinguishable from those produced by contralateral monaural tones. Attention-related modulations (ARMs) were larger in lateral than medial auditory cortex and appeared to arise in belt and parabelt auditory fields. Lateral auditory parabelt regions showed small sensory responses with evidence of a right hemispheric specialization for tone processing. Activations in lateral regions were little influenced by the acoustic properties of stimuli but showed complex intermodal interactions and were greatly enhanced by attention. The results suggest that neurons in medial auditory cortex analyze the basic acoustic features of sounds while neurons in lateral regions process more complex, behaviorally significant attributes of auditory signals.

## Methods

### Subjects

Nine subjects (aged 18–34 years, 8 male, 2 left-handed) participated after providing informed consent in accordance with the local Institutional Review Board. All subjects had normal or corrected-to-normal vision and normal hearing.

### Stimuli and tasks

Auditory stimuli were tone patterns of 750 ms duration generated by the exhaustive combination of three different 250 ms tones of different frequency. Individual tone frequencies were separated by three-semitone steps with the central tone set at 225, 900, or 3600 Hz (low, medium, or high frequency) in different blocks. Target stimuli (probability 10%) were repetitions of the previous three-tone pattern. In each block, tone intensity was fixed at either 70 or 90 dB SPL (soft or loud), and tones were delivered either to the left ear, right ear, or both ears according to a randomized design. All stimuli were presented over continuous broadband 70 dB SPL masking noise.

EPI-related scanner noise was measured with an MRI-compatible head and torso system (B&K 2260) and showed an intensity of 105 dB SPL (A-weighted) with a frequency peak at 642 Hz. The pump noise that was audible during inter-image acquisitions had an intensity of 65 dB SPL (A-weighted) and was dominated by low frequencies. Stimuli were presented through MRI-compatible electrostatic earbuds (Stax MRI-002, Stax Ltd, Saitama prefecture, Japan) that provided some attenuation of external noise over the audible frequency range. Further attenuation of ambient sounds was obtained with circumaural ear protectors (Howard Leight LM-77, Howard Leight Industries, San Diego, California, USA) that provided 25 dB of additional attenuation at 4000 Hz, 18 dB at 1000 Hz, and 6 dB at 250 Hz. Thus, the overall attenuation of external noise varied from 16–35 dB, with greater attenuation of external sounds at high frequencies.

During bimodal sequences, auditory and visual stimuli were presented asynchronously with randomized timing onsets to minimize intermodal integration. Visual stimuli were words or faces on separate blocks. In face blocks stimuli were selected from 32 black-and-white photographs of faces of eight individuals [89] depicting four different emotional expressions. In word blocks, stimuli were selected from 40 different words in ten different semantic categories (e.g., cities, plants, animals, etc.). Targets in the face blocks were successive photographs of the same individual with a different emotional expression. Targets in the word blocks were successive words belonging to the same semantic category. Responses were recorded to measure reaction times (RTs) and to permit the calculation of hit and false alarm rates. Stimulus presentation and response collection were controlled with Presentation software (NBS, Albany, CA).

### Procedure

Each subject participated in a one-hour behavioral task training session followed by high-resolution T1 structural brain imaging on a 1.5 T Philips Eclipse scanner (matrix size 256×212×256, voxel size 0.94×1.30×0.94 mm, TE 4.47 ms, TR 15 ms, flip angle 35°, field of view 240×240 mm). Thereafter, each subject participated in six 1-hr scanning sessions over a 2–6 week period: three with sparse (TR 10.8 s) and three with continuous (TR 2.9s) image acquisition. Functional imaging used a spin-echo EPI sequence (matrix size 96×96×29, 29 axial slices 4-mm thick plus 1 mm gap, voxel size 2.5×2.5×5 mm, TE 39.6 ms, flip angle 90°, FOV 240×240 mm).

Behavioral trials were presented at interstimulus intervals of 1.35 s and 1.45 s during continuous and sparse imaging, respectively, with 16 trials presented in each block. Two images were acquired per block during sparse imaging sequence and eight images were acquired per block during continuous imaging. Functional data sets from sparse and continuous imaging were analyzed separately for each subject.

We corrected for head movement using SPM5 [90]. Anatomical space analysis was used to improve the spatial resolution of functional images by coregistering individual functional images from each subject with their anatomical images and resampling each functional image into high-resolution anatomical space before analysis [91]. Functional image data were high-pass filtered with a cutoff of 0.005 Hz using polynomial detrending. Activations in voxels on the cortical surface were averaged and visualized on the spherical surface using an equal-area Mollweide projection centered on Heschl's gyrus and oriented so that the superior temporal plane lay on the equator. Average percent signal changes were calculated relative to the overall mean BOLD response for each voxel. For most analyses, mean BOLD responses associated with each block were calculated by averaging across both functional images in the sparse sampled blocks and across images 2–8 (i.e., beginning 5.8 s after block initiation) in continuous imaging sessions.

### Statistical analyses

The data were quantified by measuring the mean activation in two different grids placed over auditory cortex. The grids covered the full extent of activations on the superior temporal plane produced by auditory stimuli in all-subject averages. The medial grid contained 120 5-mm^2^ elements in an 8×15 matrix covering auditory cortex surrounding Heschl's gyrus and spanning approximately 40 mm in the medial-lateral (M-L) direction and 75 mm in the anterior-posterior (A-P) dimension (Figure 4). The lateral grid abutted the medial grid and covered lateral regions of the superior temporal gyrus. It contained 75 5 mm^2^ elements covering approximately 25 mm in the M-L dimension and 75 mm in the A-P dimension.

Grids were placed in the same locations on the left and right hemispheres after the spherically inflated right hemisphere was mirror-imaged and rigidly aligned with the left to minimize differences in surface curvature. SDAs and ARMs associated with each stimulus condition were isolated by within-subject subtractions for each imaging session. Auditory SDAs were isolated by subtracting activations in unimodal visual (UV) blocks from activations in bimodal visual-attention (BV) blocks. ARMs were isolated by subtracting BV blocks from bimodal auditory-attention (BA) blocks. Changes in activation magnitudes within blocks were examined by analyzing images 2–8 obtained during continuous imaging conditions. In addition we also analyzed all three auditory stimulation conditions (All-ASA) by subtracting unimodal visual-attention blocks from the single unimodal and two bimodal blocks containing auditory stimuli.

The spatial distribution of activation was a particular focus of interest. Distributions were analyzed over A-P and M-L dimensions within each grid. Distributions were first analyzed using uncorrected response magnitude (mean percent signal change). In addition, if there were significant main effects of stimulus features (e.g., more intense sounds produced larger activations), differences in distributions were compared after the main effects had been eliminated by normalizing the data to mean response magnitudes for each condition. This procedure isolates differences in the shape of distributions independent of overall differences in amplitude.

SDAs and ARMs were analyzed with a 9-way ANOVA for repeated measures incorporating the following factors: subjects (treated as a random factor), sparse vs. continuous imaging, tone frequency, ear of delivery, sound intensity, hemisphere, type of visual stimuli, and A-P and M-L location on the grid. All-ASA analyses were performed using a 10-way ANOVA that included three additional auditory stimulation conditions (two bimodal, and unimodal auditory) as an additional factor. Main effects and first order interactions were evaluated at the p<0.05 level after correcting the degrees of freedom using the Box-Greenhouse-Geisser correction for data covariance. Because of the large number of third- and higher-order interactions, these were evaluated using a stricter p<0.01 criterion. F-ratios and probabilities are reported for significant results and results approaching significance whereas F-ratios alone are presented for other comparisons.

### Activation maps

Spatial smoothing was applied to individual cortical surface functional image data using a 3-mm FWHM Gaussian filter [92]. Statistical F-maps for various condition comparisons were then generated by using each of the images within a block (1–2 sparse and 2–8 continuous) as an additional factor while taking into account the expected direction of BOLD activation [93]. When indicated, clustering thresholds were used [92] with hemisphere-wide Bonferroni correction to account for multiple comparisons. In order to provide improved spatial detail, all F-maps shown are fixed-effect maps (i.e., subjects are treated as a fixed effect) in contrast to the statistical analyses that treated subjects as a random effect. F-maps are used either as a mask to display only significant mean percent BOLD signal changes, or used alone to show effect significance. We also created maps that combined data from sparse and continuous imaging conditions using statistical parametric z-score maps were that obtained by converting sparse and continuous F-maps into equivalent z-score maps and then combining the z-scores at each point on the cortical surface.

## Supporting Information

Table S1 A comparison of All-ASA analyses (reported in the manuscript) and SDA analyses for data from the medial grid.
(0.06 MB DOC)

Table S2 A comparison of All-ASA analyses (reported in the manuscript) and SDA analyses for data from the lateral grid.
(0.06 MB DOC)
