# Supplementary material for: Functional Maps of Human Auditory Cortex: Effects of Acoustic Features and Attention
Source: PLoS One. 2009 Apr 13;4(4):e5183. doi: 10.1371/journal.pone.0005183 (PMC2664477; doi:10.1371/journal.pone.0005183)
Supplement: Table S1 — A comparison of All-ASA analyses (reported in the manuscript) and SDA analyses for data from the medial grid. (0.06 MB DOC) [file pone.0005183.s001.doc]

|  | *SDA* | *All-ASA* |
| --- | --- | --- |
| Sparse vs Continuous | F**_(1,8)_** = 15.79^****^ | F**_(1,8)_** = 23.75^*****^ |
| x Frequency | F**_(2,16)_** = 0.17 | F**_(2,16)_** = 0.35 |
| x Ear of Delivery | F**_(2,16)_** = 2.58 | F**_(2,16)_** = 1.25 |
| x Intensity | F**_(2,16)_** = 0.12 | F**_(2,16)_** = 0.61 |
| x Attention |  | F**_(2,16)_** = 2.55* |
| x Anterior vs Posterior | F**_(15,120)_** = 2.29^*^ | F**_(15,120)_** = 2.83^**^ |
| x Medial vs Lateral | F**_(6,48)_** = 0.97 | F**_(6,48)_** = 1.32 |
|  |  |  |
|  | *SDA* | *All-ASA* |
| Tone Frequencies | F**_(2,16)_** = 8.03^****^ | F**_(2,16)_** = 4.61^**^ |
| x Anterior vs Posterior | F**_(30,240)_** = 2.49^**^ | F**_(30,240)_** = 3.58^***^ |
| x Medial vs Lateral | F**_(12,96)_** = 2.80^**^ | F**_(12,96)_** = 5.08^***^ |
| x AP x Attention |  | F**_(60,480)_** = 1.22 |
| x AP x Image Acqu | F**_(30,240)_** = 0.92 | F**_(30,240)_** = 0.98 |
| x AP x Intensity | F**_(30,240)_** = 1.46 | F**_(30,240)_** = 1.18 |
| x AP x Hemisphere | F**_(30,240)_** = 0.92 | F**_(30,240)_** = 1.11 |
| x ML x Attention |  | F**_(24,192)_** = 1.46 |
| x ML x Image Acqu | F**_(30,240)_** = 0.99 | F**_(12,96)_** = 0.83 |
| x ML x Intensity | F**_(12,96)_** = 2.75** | F**_(12,96)_** = 1.78 |
| x ML x Hemisphere | F**_(12,96)_** = 1.26 | F**_(12,96)_** = 1.02 |
|  | *SDA* | *All-ASA* |
| Hemispheres | F**_(1,8)_** = 2.47 | F**_(1,8)_** = 2.61 |
| x Intensity | F**_(1,8)_** = 0.00 | F**_(1,8)_** = 0.01 |
| x Image Acquisition | F**_(1,8)_** = 1.76 | F**_(1,8)_** = 0.10 |
| x Frequency | F**_(2,16)_** = 0.33 | F**_(2,16)_** = 0.54 |
| x Anterior vs Posterior | F**_(15,120)_** = 2.58^*^ | F**_(15,120)_** = 2.47^*^ |
| x Medial vs Lateral | F**_(6,48)_** = 4.20^**^ | F**_(6,48)_** = 3.56^*^ |
|  | *SDA* | *All-ASA* |
| 90dB vs 70 dB | F**_(1,8)_** = 8.84^**^ | F**_(1,8)_** = 8.54^**^ |
| x Frequency | F**_(2,16)_** = 2.76 | F**_(2,16)_** = 2.67 |
| x Ear of Delivery | F**_(2,16)_** = 0;74 | F**_(2,16)_** = 1.69 |
| x Attention |  | F**_(1,8)_** = 0.05 |
| x Anterior vs Posterior | F**_(15,120)_** = 5.77^*****^ | F**_(15,120)_** = 6.38^*****^ |
| x Medial vs Lateral | F**_(6,48)_** = 1.40 | F**_(6,48)_** = 2.77^*^ |
|  | *SDA* | *All-ASA* |
| Ear of Delivery |  | F**_(2,16)_** = 2.41 |
| x Hemisphere | F**_(2,16)_** = 12.55^*****^ | F**_(2,16)_** = 13.03^*****^ |
| x Hemi x A-P | F**_(30,240)_** = 4.51^****^ | F**_(30,240)_** = 5.74^*****^ |
| x Hemi x M-L | F**_(12,96)_** = 1.32 | F**_(12,96)_** = 1.19 |
| x Frequency | F**_(1,8)_** = 1.47 | F**_(1,8)_** = 1.74 |
| x Image Acquisition | F**_(1,8)_** = 2.58 | F**_(1,8)_** = 1.90 |
| x Attention |  | F**_(2,16)_** = 0.47 |
