# Supplementary material for: Functional Maps of Human Auditory Cortex: Effects of Acoustic Features and Attention
Source: PLoS One. 2009 Apr 13;4(4):e5183. doi: 10.1371/journal.pone.0005183 (PMC2664477; doi:10.1371/journal.pone.0005183)
Supplement: Table S2 — A comparison of All-ASA analyses (reported in the manuscript) and SDA analyses for data from the lateral grid. (0.06 MB DOC) [file pone.0005183.s002.doc]

|  | *SDA* | *All-ASA* |
| --- | --- | --- |
| Sparse vs Continuous | F**_(1,8)_** = 0.63 | F**_(1,8)_** = 1.19 |
| x Frequency | F**_(2,16)_** = 0.03 | F**_(2,16)_** = 0.30 |
| x Ear of Delivery | F**_(2,16)_** = 1.88 | F**_(2,16)_** = 0.13 |
| x Intensity | F**_(1,8)_** = 0.21 | F**_(1,8)_** = 1.17 |
| x Attention |  | F**_(2,16)_** = 0.69 |
| x Anterior vs Posterior | F**_(15,120)_** = 0.70 | F**_(15,120)_** = 0.55 |
| x Medial vs Lateral | F**_(4,32)_** = 2.83 | F**_(4,32)_** = 2.83^*^ |
|  |  |  |
|  | *SDA* | *All-ASA* |
| Tone Frequencies | F**_(2,16)_** = 1.69 | F**_(2,16)_** = 0.50 |
| x Anterior vs Posterior | F**_(30,240)_** = 1.18 | F**_(30,240)_** = 0.95 |
| x Medial vs Lateral | F**_(8,64)_** = 3.46^**^ | F**_(8,64)_** = 3.03^**^ |
| x AP x Attention |  | F**_(30,240)_** = 1.04 |
| x AP x Image Acqu | F**_(30,240)_** = 1.14 | F**_(30,240)_** = 1.36 |
| x AP x Intensity | F**_(30,240)_** = 1.74 | F**_(30,240)_** = 1.36 |
| x AP x Hemisphere | F**_(30,240)_** = 0.41 | F**_(30,240)_** = 0.58 |
| x ML x Attention |  | F**_(16,128)_** = 1.87 |
| x ML x Image Acqu | F**_(8,64)_** = 0.88 | F**_(8,64)_** = 2.36^*^ |
| x ML x Intensity | F**_(8,64)_** = 0.60 | F**_(8,64)_** = 1.01 |
| x ML x Hemisphere | F**_(8,64)_** = 0.56 | F**_(8,64)_** = 0.64 |
|  | *SDA* | *All-ASA* |
| Hemispheres | F**_(1,8)_** = 9.98^**^ | F**_(1,8)_** = 7.94^**^ |
| x Intensity | F**_(1,8)_** = 0.02 | F**_(1,8)_** = 0.04 |
| x Image Acquisition | F**_(1,8)_** = 4.70^*^ | F**_(1,8)_** = 3.23 |
| x Frequency | F**_(2,16)_** = 3.53^*^ | F**_(2,16)_** = 2.10 |
| x Anterior vs Posterior | F**_(15,120)_** = 0.86 | F**_(15,120)_** = 0.94 |
| x Medial vs Lateral | F**_(4,32)_** = 2.22 | F**_(4,32)_** = 3.13^*^ |
|  | *SDA* | *All-ASA* |
| 90dB vs 70 dB | F**_(1,8)_** = 0.01 | F**_(1,8)_** = 0.04 |
| x Frequency | F**_(2,16)_** = 3.36^*^ | F**_(2,16)_** = 4.19^**^ |
| x Ear of Delivery | F**_(2,16)_** = 0.90 | F**_(2,16)_** = 1.37 |
| x Attention |  | F**_(2,16)_** = 0.27 |
| x Anterior vs Posterior | F**_(15,120)_** = 0.90 | F**_(15,120)_** = 0.84 |
| x Medial vs Lateral | F**_(4,32)_** = 1.89 | F**_(4,32)_** = 1.17 |
|  | *SDA* | *All-ASA* |
| Ear of Delivery | F**_(2,16)_** = 1.01 | F**_(2,16)_** = 0.25 |
| x Hemisphere | F**_(2,16)_** =5.69^**^ | F**_(2,16)_** =12.96^*****^ |
| x Frequency | F**_(4,32)_** = 0.68 | F**_(4,32)_** = 0.65 |
| x Attention |  | F**_(4,32)_** = 0.83 |
| x Anterior vs Posterior | F**_(30,240)_** = 1.39 | F**_(30,240)_** = 1.51 |
| x Medial vs Lateral | F**_(8,64)_** = 0.50 | F**_(8,64)_** = 0.39 |
